# Experiences of ethnic minorities engaging with mental health services during and beyond the pandemic

**DOI:** 10.1192/j.eurpsy.2025.1289

**Published:** 2025-08-26

**Authors:** Y. Fu, M. Panagioti, S. Croke, E. Stepanova

**Affiliations:** 1 University of Liverpool, Liverpool; 2 University of Manchester, Manchester; 3 University of Newcastle, Newcastle, United Kingdom

## Abstract

**Introduction:**

The pandemic exacerbated existing mental health support disparities faced by ethnic minorities in the UK. Many ethnic minorities entered care through crisis pathways, receiving more severe diagnoses than their white British counterparts. Additionally, they were 40% more likely to access mental health services via the criminal justice system. Despite these challenges, research on their evolving experiences with mental health services remains limited.

**Objectives:**

To explore the interactions between ethnic minorities and mental health services. By understanding their engagement and coping strategies, we aimed to capture how these experiences have impacted their mental health and well-being.

**Methods:**

This study was conducted in Northern England, a region with high mental health needs but limited research activity. In-depth, semi-structured interviews were held with a purposive sample of ethnic minority adults with diverse mental health conditions (ethical approval 22/WS/0164).

Two independent researchers conducted interviews remotely or in person between March and September 2023, with consent confirmed before each interview. The topic guide, co-produced and piloted with an advisory group of ethnic minority individuals, carers, and clinicians, focused on service engagement, support experiences, coping strategies during the pandemic, and suggestions for improvement. Data were analysed using a framework approach, with themes and subthemes categorized in a matrix for each transcript. Two researchers independently double-coded a sample of interviews to ensure validity, with the team and advisory group reviewing and finalizing the analytical framework.

**Results:**

Thirty-two ethnic minority individuals were interviewed, revealing five key themes: barriers to managing mental health; limited engagement with health services; preference for community support; reliance on community support during service interruptions in the pandemic; and the need for service-community collaboration. Cultural stigma often led to fear and reluctance to seek support, and participants struggled with non-culturally sensitive health services. Instead, they preferred community-based support, which persisted during the pandemic despite service disruptions. Participants advocated for collaboration between mental health services and ethnic minority communities to enhance cultural understanding and patient-centred care.

**Conclusions:**

Ethnic minorities with mental health conditions face significant challenges in accessing and engaging with services. Addressing these issues requires integrating culturally sensitive approaches into existing frameworks, achieved through collaborations with ethnic minority communities to better understand their unique contexts. Incorporating cultural considerations into service delivery can enhance engagement and improve outcomes for diverse populations.

**Disclosure of Interest:**

None Declared

